# Supplementary material for: Investigating the potential of packed cell volume for deducing hemoglobin: Cholistani camels in perspective
Source: PLoS One. 2023 May 16;18(5):e0280659. doi: 10.1371/journal.pone.0280659 (PMC10187907; doi:10.1371/journal.pone.0280659)
Supplement: Attachment — Submitted filename: Response to Reviewers.docx [file pone.0280659.s001.docx]

**Rebuttal for Reviewer’s Comments for PONE-D-22-35645**

| **Reviewer’s Comments/suggestions** | **Action Taken/Justification** |
| --- | --- |
| **REVIEWER 1** | |
| **Abstract** The section is well written, however, the statement “A good agreement between HbD and CHb and there was no proportional bias on the distribution of data around the mean difference line (Mean= 0.1436, 95%CI= 3.00—2.72).” needs to be rephrased. | The statement mentioned by the reviewer is, in fact, the result of Bland-Altman agreement analysis.  The said sentence has been rephrased in the amended draft as per the recommended pattern of giving results for Bland-Altman test, please |
| **Introduction**  The first few lines of the introductory paragraph seem to simply define the PCV which is not required, rather the authors must through light on the functional role of PCV and its existing relationship with Hb with reference to the species under study. Authors must cite the research showing the existing relationship of PCV and Hb as deduced in human and other animal subjects. | This is true that the introductory two paragraphs were much about PCV alone. Both first two paragraphs have been merged as one and briefed up. They will highlight the reader towards the PCV and its role.  As the studies regarding interrelationship between Hb and PCV are almost none. Hence, for sake of devising research hypothesis, studies conducted mainly on humans have been incorporated, please. |
| **Methodology**  The methodology employed is robust, however information about following points is lacking Time of blood collection, type and volume of syringe used for blood collection, type of anti-coagulant used, storage temperature etc. The sample size is ambiguous. Have you sub classed the data in terms of gender and age? How many replications were performed? | The brief about number of samples, type of sampling, method of blood collection etc have been incorporated in the amended draft, please. |
| **Statistical Analyses**  As per the authors, the data did observe the normal distribution and use of non-parametric tests is understood. However, when the going for regression analysis, have you gone for data transformation? and what type of post hoc test was performed? | Our data was non-normal. Hence, non-parametric test of Mann-Whitney U test was implied.  Furthermore, no data transformation was carried out for linear regression. Because the “Linear regression analysis, which includes t-test and ANOVA, does not assume normality for either predictors (IV) or an outcome (DV)”. (Reference: Research Data Services, University of Virginia Library). |
| **Results**   - Use either Colistani camels or cattle in both of the tables 1 and 2. - In table 2, the information is little and needs more clarification. Authors must add the equations of both the regression analysis performed (HbD vs PCV and HbD vs HbC) and later on show the changes in adjusted r2. | - The tables have been corrected, please. - Table 2 already contains regression equations for all relevant attributes, please. |
| **Discussion**   - The first paragraph of the discussion section is giving information which is not supported by the results of the study. You should limit such information to the introduction portion. - There is a very little information about the hematology of camels in the discussion section so the authors must include good number of comparisons citing the previous reports on other camel species. - Results of the study have been very poorly discussed. The authors must discuss their results, and provide valid grounds such as intrinsic physiological/species- specific, gender, age, body size, basis of the significant differences observed. Furthermore the study needs validation in terms of the statistical methodology which is being recommended and widely used in the studies with similar reports. | - The introductory paragraph has been briefed up and unnecessary text has been deleted - Owing to paucity of literature on interrelationship of PCV and Hb in camels, comparisons have been made with human studies - Results have been compared briefly with those of humans due to paucity of such work in livestock. Furthermore, results of Bland-altman and correlation coefficient have been elaborated in amended draft, please. |
| **REVIEWER 2** | |
| Are the results found for PCV values in the normal range for camels? | Yes. The mean values for HbD and PCV were within the normal reference range for camels. Similar has been mentioned in Results section of the amended draft. |
| Many references from the discussion are from cattle. It would be nicer to have more information about camels in the discussion. | Appropriate references for camels have been incorporated. |
| Other doubt is about the term: “pen-side”. Usually, when we use “pen-side” analysis is for something that could be done at the moment an animal is examined. In this case, since it is necessary to go to a laboratory (or at least, to have the hematocrit done) to estimate the hemoglobin concentration, can we say it is pen-side? | This is a very wisely and intellectually drawn concern of the reviewer. In this regard, it is submitted that it is true that the blood needs to be taken to the laboratory for conducting PCV or the microhematocrit machine. However, the microhematocrit machine- a small portable, cheap machine can be taken to the field and with a single drop of blood, PCV can be deduced. This is why it has been termed as ‘pen-side’.  It is further justified that the title of the research project for which research grant has been awarded by the Pakistan Science Foundation also contains ‘pen-side’ in it. |
| **Materials and methods** More information about the camels could be given, like: are they from a specific breed? What are they purpose? The camels were from how many herds? | Corrected and appropriate information has been added in amended draft, please. |
| It would also be nice to explain the age groups. When is a camel considered adult? With what age? | Age groups have been defined in amended draft, please. |
| All the animals were being reared under similar management and feeding conditions of pastoral- ism. Split-herding is normally exercised for livestock by the pastoralists, according to which the young ones (calves in this case) are kept at their pens near the “Tobas” (natural or man-mad water reservoirs of the desert), while the adults are sent for grazing till night-time (Farooq et al., 2010).” Is this about the camels? Because it is the same explanation in the cattle article. | The error has been corrected in amended draft. Camel herds are grazed as per rotational herding. Detail has been given now in Methodology, please. |
| Regarding anemia, the reference of Grace et al may be replaced by that for camels. | The reference for cattle (Grace et al., 2007) has been replaced with Saleh et al., (2009) related to camel anemia. |
| Reference corrections | All references in text and in list have been corrected.  Ahamd et al reference has been mentioned as 2022a and 2022b. |
